# Affective-Cognitive Behavioral Therapy for Fibromyalgia: A Randomized Controlled Trial

**DOI:** 10.1155/2012/937873

**Published:** 2011-09-07

**Authors:** Robert L. Woolfolk, Lesley A. Allen, Jeffrey T. Apter

**Affiliations:** ^1^Department of Psychology, Rutgers University, New Brunswick, NJ 08901, USA; ^2^Department of Psychology, Princeton University, Princeton, NJ 08544, USA; ^3^Department of Psychiatry, UMDNJ—Robert Wood Johnson Medical School, Piscataway, NJ 08854, USA; ^4^University Medical Center at Princeton, Princeton, NJ 08540, USA

## Abstract

A randomized controlled trial was conducted to assess the efficacy of an individually administered form of cognitive behavioral treatment for fibromyalgia. In an additive design, 76 patients diagnosed with fibromyalgia were randomly assigned to either the experimental treatment (affective-cognitive behavioral therapy, 10 individual sessions, one per week) administered concurrently with treatment-as-usual or to an unaugmented treatment-as-usual condition. Statistical analysis conducted at the end of treatment (3 months after the baseline assessment) and at a followup (9 months after the baseline assessment) indicated that the patients receiving the experimental treatment reported less pain and overall better functioning than control patients, both at posttreatment and at followup. The implications of these findings for future research are discussed.

## 1. Introduction

Fibromyalgia (FM) is a prevalent and disabling syndrome. It is characterized by widespread musculoskeletal pain, multiple tender points, sleep disturbance, fatigue, and stiffness [1, 2]. The prevalence of FM has been estimated to be about 2% of the population [2]. Patients meeting criteria for FM have been shown to overuse health care services and experience high rates of disability [3–5]. 

At present, FM appears to be extremely challenging to treat [6]. Although some pharmacological and nonpharmacological treatments have produced moderate benefits, no intervention has yet been demonstrated capable of generating clinically significant improvement in the majority of FM patients [6]. The controlled clinical trial literature suggests that pharmacological agents provide some relief to FM patients, though the magnitude of these effects is modest [7, 8]. Psychosocial interventions also have shown some promise in alleviating FM symptoms, with exercise programs and cognitive-behavioral treatments appearing most potent [8, 9]. Notwithstanding, empirical reviews of the efficacy of cognitive-behavioral treatment (CBT) for FM have revealed mixed results, some showing low-to-medium effect sizes [9, 10], others showing no effect [11]. Because, to date, CBT for FM has been administered in groups, the efficacy of *individually administered* CBT for FM has not been assessed within a controlled experimental design. We hypothesized that an individually administered, intensive, and individualized CBT treatment would achieve more powerful effects than previous group-administered CBT. 

We developed an individually administered (CBT) for FM that includes relaxation training, activity regulation, facilitation of emotional awareness, cognitive restructuring, and interpersonal communication training. The elicitation and exploration of affect is an approach rarely used in CBT [12]. We, however, have found this component to be a powerful clinical tool with patients who cannot or do not willingly access and experience emotion, indeed so powerful that we have sometimes labeled our approach affective-cognitive behavioral therapy (ACBT) [13]. In this investigation, we hypothesized that ACBT would reduce pain intensity and improve other symptomatology over and above the effects of treatment as usual in patients with FM.

## 2. Materials and Methods

### 2.1. Study Design

We conducted a randomized, controlled treatment trial, using an additive design [14], in which patients diagnosed with FM received 1 of 2 treatments: (1) 10 weekly sessions of individually administered ACBT in addition to treatment-as-usual (TAU) or (2) TAU alone. Participants were assessed three times during the course of the study: at baseline, 3 months after baseline (posttreatment), and 9 months after baseline (followup).

### 2.2. Study Population and Settings

Participants were referred to the study by their treating rheumatologists. Men and women, ages 18 to 70, who met ACR criteria for FM, as diagnosed by their rheumatologists and confirmed by a medical history review, were eligible for the study. Individuals manifesting any of the following were excluded from the study: pain from traumatic injury or structural or regional rheumatic disease, rheumatoid arthritis, inflammatory arthritis, autoimmune disease, unstable medical or psychiatric illness, active suicidal ideation, a history of psychosis, current psychoactive substance dependence, or a medication regimen that had not been stable for at least 2 months prior to baseline. Women who were pregnant or attempting to conceive also were excluded from the study. Participation in psychotherapy concurrent with the period between the baseline and posttreatment appointment, which occurred 3 months after baseline, was not permitted.

The study took place in an academic medical clinic at Robert Wood Johnson Medical School (RWJMS). The study was approved by RWJMS's institutional review board. Written informed consent was obtained from all participants.

### 2.3. Treatment Conditions

The ACBT is a 10-session, individually-administered, manualized intervention designed for patients with functional somatic symptoms. The treatment, which we developed, is described in detail elsewhere [13]. The treatment manual allows for adaptation and adjustment to the individual pattern of symptoms and life situations presented by the patients.

### 2.4. Randomization and Masking

Participants were randomly assigned to ACBT + TAU or TAU using a computer-generated random number sequence. Neither blocking nor stratification was used. Study personnel administering questionnaires were masked to participants' treatment condition.

### 2.5. Initial Assessment

Participants were assessed at baseline, just before treatment began. Demographic characteristics and baseline levels of the outcome measures (described below) were assessed. The Hollingshead four-factor index was employed to measure participants' socioeconomic status [15]. It is a widely used measure calculated from an individual's (and his/her working spouse's, if applicable) educational background and occupational history.

### 2.6. Outcome Measures

The primary outcome measure was a 10 cm visual analog scale of pain (VAS) anchored at its lowest point by the expression “no pain” and at its highest point by the phrase “very severe pain.” Participants were asked to rate their level of pain over the preceding seven days. The VAS has been used widely in FM clinical trials to measure pain severity and appears to be sensitive to change [16]. 

Secondary measures included the Medical Outcomes Study (MOS) SF-36 Physical Functioning Scale (MOS-PF), the Chronic Pain Self-Efficacy Scale (CPSE), the Beck Depression Inventory (BDI), and the Beck Anxiety Inventory (BAI). Physical functioning was assessed with the physical functioning subscale of the MOS SF-36, a 36-item self-report questionnaire assessing various aspects of quality of life. The MOS SF-36 has been validated across a wide range of conditions including fibromyalgia [17, 18]. Self-efficacy for pain management was assessed with the pain management subscale of the Chronic Pain Self-Efficacy Scale (CPSE) [19]. Current level of depression was assessed with the Beck Depression Inventory (BDI), a 21-item self-report questionnaire measuring various aspects of depression. The BDI has been used widely in the depression and fibromyalgia literatures and is considered psychometrically sound [20, 21]. Current level of anxiety was measured with the Beck Anxiety Inventory (BAI). The BAI is a 21-item self-report scale assessing the affective, cognitive, and physical symptoms of anxiety which has demonstrated sound psychometric properties [22, 23].

Because patients' expectations for treatment outcome may be associated with response to treatment [24], they were assessed with the Expectation Rating Scale [25]. The Expectation Rating Scale is made up of three statements to which patients respond by placing a mark on a 10 cm VAS [25].

### 2.7. Statistical Analysis

**Figure 1:**
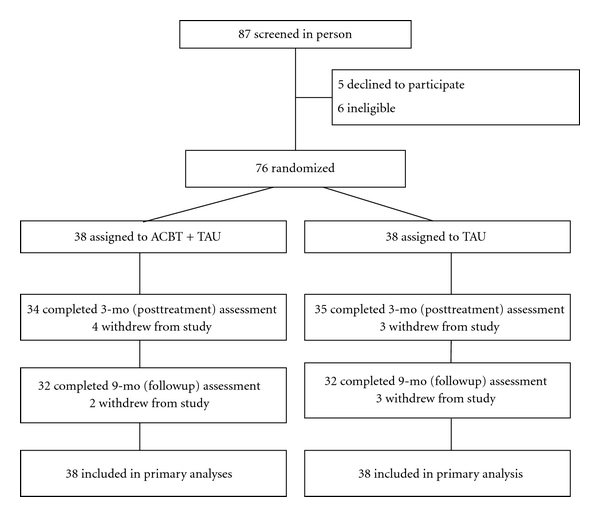
Flow of participants through the study.

Differences between groups on baseline characteristics were tested using unpaired *t-*tests for continuous variables or *χ* ^2^ tests for categorical variables. An intent-to-treat approach, based on data from all randomized participants, was used in all analyses. The treatment condition (ACBT + TAU or TAU) served as the independent variable contrast in all analyses, in what is typically referred to as an additive design, in which both levels of the independent variable possess a common element to which, in one group, a putatively therapeutic agent is added [14]. Groups were compared on the primary and secondary outcome variables at the posttreatment and at follow-up appointments, 3 months and 9 months after baseline, respectively. In all, 12 participants were lost to attrition (see Figure 1). Missing data were imputed via the last observation carried forward method. Bonferroni's correction was used to control for the effect of multiple comparisons on overall experiment-wise error rate, which was set as *P* < .05. All tests of statistical significance were 2-tailed, and all statistical analyses were performed using SAS, version 8 (SAS Institute Inc, Cary, NC).

## 3. Results

There were no significant differences on any baseline characteristics between the two treatment groups (Table 1), suggesting that outcome findings related to treatment were not confounded by any demographic variable. Most participants were middle-aged women who had experienced widespread pain for an average of 11.5 years. 

**Figure 2:**
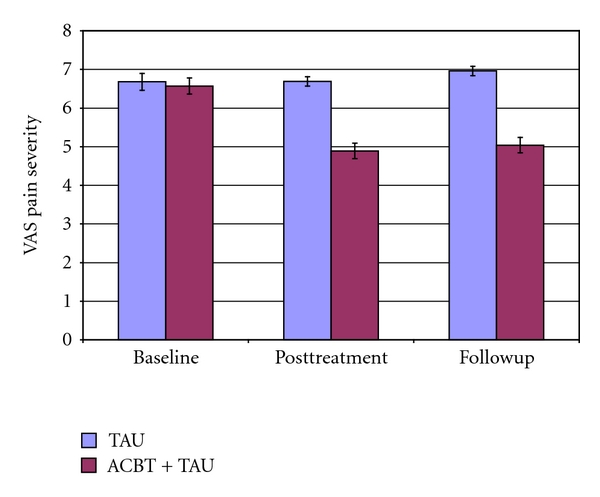
Mean and standard error of the mean of the Visual Analogue Scale (VAS) for pain severity TAU indicates treatment as usual. ACBT indicates affective cognitive-behavioral treatment.

A one-way analysis of covariance with one fixed effect (ACBT + TAU versus TAU), using baseline scores as the covariate, was conducted on the primary outcome measure (VAS) at posttreatment. The main effect for treatment was highly significant, *F*(1,73) = 45.94, *P* < .0001, Hedges's *g* = 0.90, with patients receiving ACBT + TAU indicating less pain than those receiving TAU. At followup the difference between treatment conditions continued to be highly significant, *F*(1,73) = 52.83, *P* < .0001, Hedges's *g* = 0.95 (see Figure 2).

The data were also examined from the perspective of clinical significance, using the criterion of 30% improvement. At posttreatment 25 patients (65.8% of the intent-to-treat sample) in the ACBT + TAU group, showed at least 30% improvement from baseline on the VAS, whereas only 2 patients (5.2%) in the TAU group improved by 30%. At followup 24 patients in the ACBT + TAU group (63.2%) were at least 30% improved from baseline on the VAS, whereas only one assessed patient (2.6%) in the TAU group continued to be improved by 30%.

**Figure 3:**
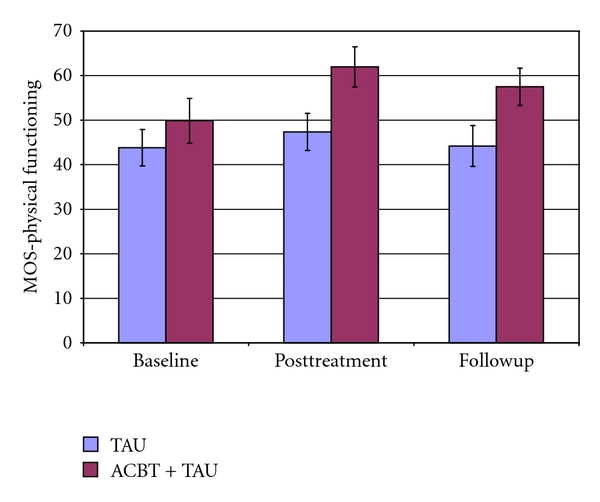
Mean and standard error of the mean of the MOS-physical functioning scale TAU indicates treatment as usual. ACBT indicates affective cognitive-behavioral treatment.

**Figure 4:**
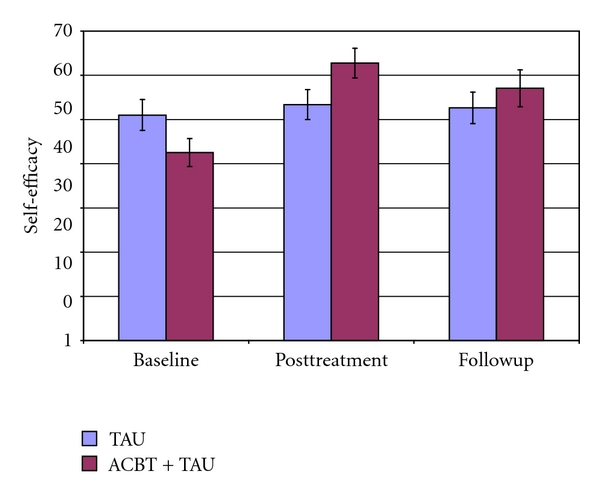
Mean and standard error of the mean of the self-efficacy for pain management scale. TAU indicates treatment as usual. ACBT indicates affective cognitive-behavioral treatment.

In Table 2, a summary is presented of all analyses of primary and secondary dependent variables. The overall pattern of results shows a relatively strong effect for the ACBT upon pain in FM, an effect that continues at followup. Significant but weaker effects were discovered for all the secondary targets at posttreatment, but a Bonferroni correction would have rendered the effect on the CPSE pain management scale less than significant at followup, when correcting for multiple comparisons (see Figures 3 and 4). 

## 4. Discussion

Our findings suggest that an intensive individually-administered ACBT produces significant improvement in self-reported pain in FM. The treatment's impact on self-reported physical functioning, self-efficacy, depression, and anxiety was statistically significant but smaller. Our data are consistent with recent reviews that have found CBT to be perhaps the most effective psychosocial treatment for FM [9]. The effect size found for ACBT on VAS pain severity was both large and durable compared to those reported in other studies examining CBT, although findings on the other study variables were more or less in line with results of earlier research. The VAS is often considered to be the instrument of choice in studying treatment of FM patients [16], although there is enough variability in the way the VAS is presented to study patients, for example, differing anchor points, that comparisons across studies are rendered somewhat problematic. If we simply look within our own sample nonparametrically, using a 30% improvement on the VAS as the criterion, the ACBT augmentation of TAU is clinically quite significant, given that TAU was almost entirely without benefit in our sample. Several other studies of treatment for FM have found TAU to yield no clinically significant improvement [26, 27]. 

Given the structure of the experimental design, however, we cannot infer with certainty that factors unique to ACBT were causal elements in the observed changes or that ACBT administered without TAU would be an effective treatment. What we observed was the successful augmentation of TAU by ACBT. Given that generally, and especially in our study, TAU is not an efficacious treatment for FM, our findings suggest that future research should examine the potential utility of treatments such as the one evaluated here. One question that could be addressed is whether additional ACBT sessions, either in the form of an extended treatment or “booster sessions” occurring in the months following the initial intervention, would yield greater therapeutic impact. 

There are a variety of reasons why ACBT may have been especially beneficial to our patients. Because each of our patients received 10 individual sessions with the same therapist, perhaps a somewhat stronger bond may have developed between patient and therapist than is often seen in group-administered CBT. The use of extensive relaxation training and exploration of emotions gives our treatment [13] some of the ambience of standard psychotherapy as it is practiced in the generic clinical arena, rather than the somewhat psychoeducational feeling that group-administered CBT can sometimes possess. Whether relationship factors per se added to the therapeutic power or simply inclined participants to indicate more symptom relief is impossible to say but raises questions that could be systematically examined in subsequent research. The failure to find higher expectations for treatment among ACBT + TAU patients suggests that the treatment effect was not due to mechanisms implicated in the response to placebos.

The durability of our treatment effect upon pain may have had to do with the more intensive, individualized treatment that individually administered sessions can provide. The very use of relaxation training throughout treatment and the strong emphasis given to it as a valuable stress management skill that should be regularly applied in one's life and be a permanent part of one's coping repertory may give our patients a tool that is effective in reducing the discomfort associated with FM. Whether the component of our treatment that places patients in closer touch with emotions that are often suppressed or denied is a factor in treatment efficacy is a question to be answered in future research. From a practical clinical standpoint, it would appear that our approach to cognitive behavioral therapy, ACBT, can be individually administered to FM patients with some likelihood of improving their symptoms.

## 5. Conclusion

An individually administered affective-cognitive behavioral treatment resulted in sustained improvement in pain and related symptomatology in a sample of patients with FM who had been referred for treatment by their rheumatologists. Additional research is needed to replicate our findings and to explore some questions raised. Is intensive, individually administered CBT a more powerful treatment for FM than treatment provided in patient groups? Are the factors that are stressed in our treatment, creating high competence in relaxation methods and emphasizing the patient's emotional self-awareness, important to success in the psychosocial treatment of FM?

## Figures and Tables

**Table 1:** Baseline characteristics of the participants*.

	ACBT + TAU	TAU	*P* value
	*n* = 38	*n* = 38	
Age, mean (SD), y	47.79 (9.28)	50.21 (10.14)	n.s.
Female, no. (%)	34 (89.47)	33 (86.84)	n.s.
Race/ethnicity, no. (%)			
White	30 (78.95)	28 (73.68)	n.s.
African american	2 (5.26)	0 (0.00)	
Hispanic	3 (7.89)	6 (15.79)	
Other	3 (7.89)	4 (10.53)	
Education, no. (%)			
Graduate degree	10 (26.32)	6 (15.79)	n.s.
College degree	10 (26.32)	12 (31.58)	
Some college	9 (23.68)	13 (34.21)	
High school or less	9 (23.68)	7 (18.42)	
Married, no. (%)	19 (50.00)	21 (55.26)	n.s.
Employed, no. (%)	16 (42.11)	21 (55.26)	n.s.
Hollingshead SES, mean (SD)	47.51 (10.20)	49.61 (9.61)	n.s.
Expectation Rating Scale, mean (SD)	17.20 (5.21)	16.09 (6.86)	n.s

ACBT indicates affective cognitive behavioral therapy, TAU indicates treatment as usual, Hollingshead SES indicates Hollingshead socioeconomic status scale score.

*Data are presented as No. (%) unless otherwise indicated.

**Table 2:** Summary of outcomes.

Outcomes		*F*	*P*	Hedges' *g *
Visual analogue scale for pain severity	Posttreatment	45.94	<.0001	0.90
	Followup	52.83	<.0001	0.95

MOS SF-36 physical functioning	Posttreatment	13.25	<.0005	0.35
	Followup	9.89	<.0024	0.28

Self-efficacy for pain management	Posttreatment	10.42	<.0019	0.65
	Followup	4.13	<.0459	0.40

Beck depression inventory	Posttreatment	11.03	<.001	0.56
	Followup	15.70	<.0002	0.60

Beck anxiety inventory	Posttreatment	11.79	<.001	0.45
	Followup	12.04	<.0009	0.62
